# The chemical reactions in electrosprays of water do not always correspond to those at the pristine air–water interface†

**DOI:** 10.1039/c8sc05538f

**Published:** 2018-12-21

**Authors:** Adair Gallo, Andreia S. F. Farinha, Miguel Dinis, Abdul-Hamid Emwas, Adriano Santana, Robert J. Nielsen, William A. Goddard, Himanshu Mishra

**Affiliations:** King Abdullah University of Science and Technology (KAUST) Saudi Arabia himanshu.mishra@kaust.edu.sa; Water Desalination and Reuse Center (WDRC) Saudi Arabia; Division of Biological and Environmental Sciences (BESE) Saudi Arabia; KAUST Catalysis Center (KCC) Saudi Arabia; Imaging and Characterization Core Laboratory Thuwal 23955-6900 Saudi Arabia; Materials and Process Simulation Center, California Institute of Technology Pasadena CA 91125 USA

## Abstract

The recent application of electrosprays to characterize the air–water interface, along with the reports on dramatically accelerated chemical reactions in aqueous electrosprays, have sparked a broad interest. Herein, we report on complementary laboratory and *in silico* experiments tracking the oligomerization of isoprene, an important biogenic gas, in electrosprays and isoprene–water emulsions to differentiate the contributions of interfacial effects from those of high voltages leading to charge-separation and concentration of reactants in the electrosprays. To this end, we employed electrospray ionization mass spectrometry, proton nuclear magnetic resonance, *ab initio* calculations and molecular dynamics simulations. We found that the oligomerization of isoprene in aqueous electrosprays involved minimally hydrated and highly reactive hydronium ions. Those conditions, however, are non-existent at pristine air–water interfaces and oil–water emulsions under normal temperature and pressure. Thus, electrosprays should be complemented with surface-specific platforms and theoretical methods to reliably investigate chemistries at the pristine air–water interface.

## Abstract 2

This contribution explains the origin of dramatic rate accelerations in chemical reactions taking place in/on aqueous electrosprays. We combine experiments with electrosprays and proton-nuclear magnetic resonance with quantum mechanics to systematically decouple genuine interfacial effects from non-equilibrium conditions.

## Graphical abstract

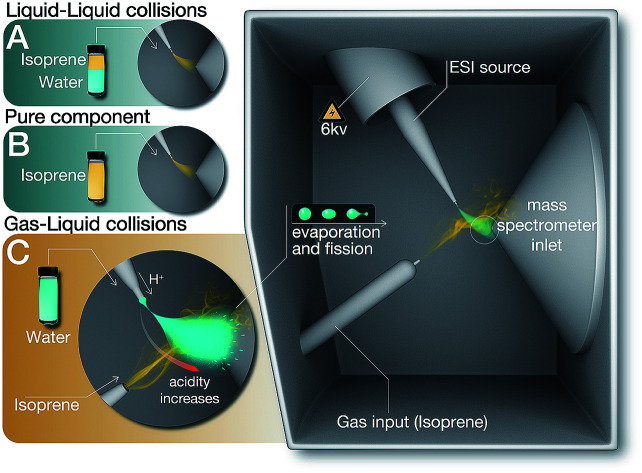

The air–water interface plays a critical role in numerous natural and applied contexts, such as atmospheric chemistries,^1–3^ precipitation,^4^ spray coatings,^5^ and materials synthesis.^6,7^ Indeed, it has been hypothesized that microdroplets generated during the splashing of waves in oceans could have been the chemical reactors leading to the origin of life.^8–10^ Despite its ubiquity and importance, a variety of fundamental phenomena at the air–water interface remain incompletely understood, such as the specific adsorption of ions^11–13^ and chemistries therein.^10,14–20^ The interfacial region, with a typical thickness *δ*_o_ ≈ 0.5 nm, separates the gas-phase (vapor) from the condensed phase (water), two drastically different regions in terms of hydration – reactions spontaneous in one phase are forbidden in the other.^21^ In fact, the chemical activities of species at the air–water interface can depart significantly from those in the bulk, as has been demonstrated by surface-specific techniques, including vibrational second harmonic generation and sum frequency generation,^20,22,23^ and polarization-modulated infrared absorption reflection spectroscopy,^10,13^ and indirect approaches, including NMR^24^ and confocal fluorescence microscopy.^25^ Even though vibrational spectroscopy-based techniques report directly on thermodynamic properties of the air–water interface, they suffer from interpretational ambiguities and limitations due to low signal-to-noise ratios.^26–32^ Thus, new techniques with higher sensitivity and unambiguous response are needed to help resolve the poorly understood features of the air–water interface while providing benchmarks to judge previous interpretations.^33^ In this work, we assess the application of electrospray ionization mass spectrometry (ESIMS) to unravel the thermodynamic properties of pristine air–water interface (Henceforth, we will use the qualifier ‘pristine’ to refer to the air–water interface that is not under the influence of any external sources/agents, such as electrical voltage or a drying gas).

In the recent years, ESIMS, which has been widely used to characterize ionic/molecular species in polar/apolar solvents,^34^ has been adapted to investigate the pristine air–water interface. In the standard configuration, ESIMS experiments entail the formation of electrosprays by the application of electrical potential and/or pneumatic pressure leading microscale droplets with excess electrical charge; those microdroplets pass through a glass/metallic capillary maintained at elevated temperature (∼473 K) to evaporate the solvent and facilitate the mass spectrometric detection of analytes downstream.^34–38^ In the experiments designed to investigate chemistries at the air–water interface, electrosprays containing one or more reactant(s) are intersected with gases or other electrosprays containing other reactant(s) followed by mass spectrometric detection. For instance, using this platform, thermodynamic properties of the pristine air–water interface have been explored under ambient conditions, including the relative concentrations of interfacial hydronium and hydroxide ions and their activities^39–43^ leading to interpretations that have elicited scientific debate.^11,12,17–19,44,45^ Further, by intersecting electrosprays of pH-adjusted water with gaseous organic acids,^40^ isoprene,^41^ and terpenes,^40,41,46,47^ researchers observed instantaneous protonation (<1 ms), and in some cases oligomerization of organics, which led them to conclude that as the bulk acidity of water approaches pH ≤ 3.6, the pristine air–water interface behaves as a superacid. While a clear understanding of the emergence of the putative superacidity at the air–water interface is unavailable, we note that in the condensed phase proton-catalyzed oligomerization of isoprene (or olefins in general) requires 60–80% concentrated H_2_SO_4_ solutions (pH < −0.5).^48^ Similar rate enhancements in aqueous electrosprays have also been observed for the syntheses of abiotic sugar phosphates,^15,49^ the Pomeranz–Fritsch synthesis of isoquinoline,^50^ the reaction between *o*-phthalaldehyde and alanine,^51^ and the ozonation of oleic acid,^52^ among others.^16,53^ Herein, we assess the relationships between the chemistries observed in aqueous electrosprays to those at pristine air–water interfaces; we also seek to decouple the factors that contribute to the mechanisms underlying reported dramatic rate enhancements by addressing the following questions:

(i) Do aqueous electrospray-based platforms report on thermodynamic properties of the pristine air–water interface?

(ii) Do accelerated reactions in aqueous electrosprays arise only from the significant enhancement of the hydrophobe–water (air–water) interfacial area? If yes, the mechanisms underlying the dramatic rate enhancements therein should be insightful in explaining the accelerated organic reactions in oil–water emulsions also referred to as ‘on-water’ catalysis.^54–57^

(iii) Are the rate accelerations in aqueous electrosprays driven solely by the non-equilibrium conditions therein, especially the enhanced concentration of reactants in the micro-/nano-droplets due to the evaporation of water?^58–61^

(iv) Are gas-phase reactions implicated in the acceleration of chemical reactions in aqueous electrosprays?^36,44,50,62–64^

To address those questions, we investigated the oligomerization of isoprene by proton nuclear magnetic resonance (^1^H-NMR), a non-invasive technique, as a complementary platform to the ESIMS. Questions (i and ii) were addressed by comparing the effects of enhancing the water–hydrophobe interfacial area in both liquid–vapor and liquid–liquid systems; questions (iii and iv) were addressed by varying the capillary voltages, ionic strengths of the aqueous solutions electrosprayed and intersected with gas-phase isoprene, and ^1^H-NMR analysis of condensed vapor from the electrosprays. To highlight the role of hydration in electrosprays, we performed quantum mechanical calculations employing density functional theory (M06 flavor).

## Materials and methods

In our experiments, we used isoprene (99% purity from Sigma-Aldrich), Mili-Q deionized water (18 MΩ m resistivity), D_2_O (99.9% purity from Sigma Aldrich), ethanol (absolute from Merck Millipore), acetone (HPLC standard from VWR Chemicals), NaCl (>99% purity from Sigma Aldrich), HCl (36.9% concentration from Fisher Scientific), DCl (35% concentration 99% deuterium purity from Sigma Aldrich), and NaOH (>97% purity from Sigma Aldrich) to adjust the pH and ionic strengths.

### ESIMS

All experiments were conducted in a commercial Thermo Scientific – LCQ Fleet electrospray ionization mass spectrometer in the positive ion mode, where a DC potential of 6–8 kV was applied to the needle, the tube lens voltage was 75 V, the sheath gas flow rate was 10 arb, the pressure was 1.2 torr at the convection gauge and 0.8 × 10^−5^ torr at the ion-gauge, the flow rates of analytes were controlled by a calibrated syringe pump and ranged between 1–10 μL min^−1^, the distances from the ion source and the inlet to the mass spectrometer were ∼2 cm, and the distance between the electrospray and the tube ejecting isoprene was 1 cm.

### ^1^H-NMR

All NMR spectra were acquired using a Bruker 700 AVANAC III spectrometer equipped with a Bruker CP TCI multinuclear CryoProbe (BrukerBioSpin, Rheinstetten, Germany); Bruker Topspin 2.1 software was used to collect and analyze the data. We transferred 100 μL of the (A1) samples into 5 mm NMR tubes, followed by 600 μL of deuterated chloroform (CDCl_3_). The ^1^H-NMR spectra were recorded at 298 K by collecting 32 scans with a recycle delay of 5 s, using a standard 1D 90° pulse sequence and standard (zg) program from the Bruker pulse library. The chemical shifts were adjusted using tetramethylsilane (TMS) as an internal chemical shift reference. The (A) samples and a sample of as-purchased isoprene (B) were prepared by transferring 100 μL of each to 5 mm NMR tubes, and then adding 550 μL of deuterated water D_2_O to the NMR tubes. The ^1^H-NMR spectra were recorded by collecting 512 scans with a recycle delay time of 5 s, using an excitation sculpting pulse sequence (zgesgp) program from the Bruker pulse library. The chemical shifts were adjusted using 3-trimethylsilylpropane sulfonic acid (DSS) as an internal chemical shift reference. The free induction decay (FID) data were collected at a spectral width of 16 ppm into 64 k data points. The FID signals were amplified by an exponential line-broadening factor of 1 Hz before Fourier transformation.

### Computational methods

To gain molecular-level insights into the protonation and oligomerization of the isoprene in our experiments, we performed (and benchmarked) DFT calculations at the following levels: (A) geometry and transition state optimization with M06/6-311G* followed by single point calculations with a larger basis set (6-311++G**);^65^ (B) geometry and transition state optimization with M06/6-311++G**; (C) geometry and transition state optimization with M06/6-311++G**, followed by single point calculations with CCSD(T).^66^ We calculated the internal reaction coordinate (IRC) along the potential energy surface connecting adduct (A), transition state (TS), and product (P). Frequency calculations of the optimized geometries yielded Hessians with zero and one imaginary frequency, respectively, for the minima and the transition state structures. The force constants from the frequency calculations on the TS structures were subsequently used to calculate the IRC path along the forward and backward direction of the transition vector, connecting transition state to the minima.^67,68^ The vibrational frequencies from the Hessians were also used to provide the zero-point energies and vibrational contributions to the enthalpies and entropies. The free energies of isoprene at 1 atm were calculated using statistical mechanics for ideal gases.

### Molecular simulations

Born–Oppenheimer molecular dynamics (BOMD)^66^ simulations were carried out with the M06 functional and 6-311G(d,p) basis set in the NVT ensemble coupled with a Nose–Hoover thermostat^69^ at 298 K. The total simulation times were 5 ps with 1 fs timesteps.

We used the Gaussian 09 software package^70^ to perform these calculations and simulations.

## Results

We investigated chemical reactions between pH-adjusted water and isoprene (C_5_H_8_, 2-methyl-l,3-butadiene, MW = 68 amu, and solubility in water, *S* = 0.7 g L^−1^ at normal temperature and pressure (NTP): 293 K and 1 atm). We chose to examine reactions of isoprene because (i) we wanted to reproduce previous experimental results to ensure a clear comparison, (ii) isoprene is an important biogenic gas whose fate in the atmosphere is not completely understood,^1,41,71,72^ and (iii) we could investigate chemistries in electrosprays and emulsions by taking advantage of the low boiling point of isoprene (*T*_b_ = 307 K) and the high vapor pressure at NTP (*p* = 61 kPa).^73^

**Fig. 1:**
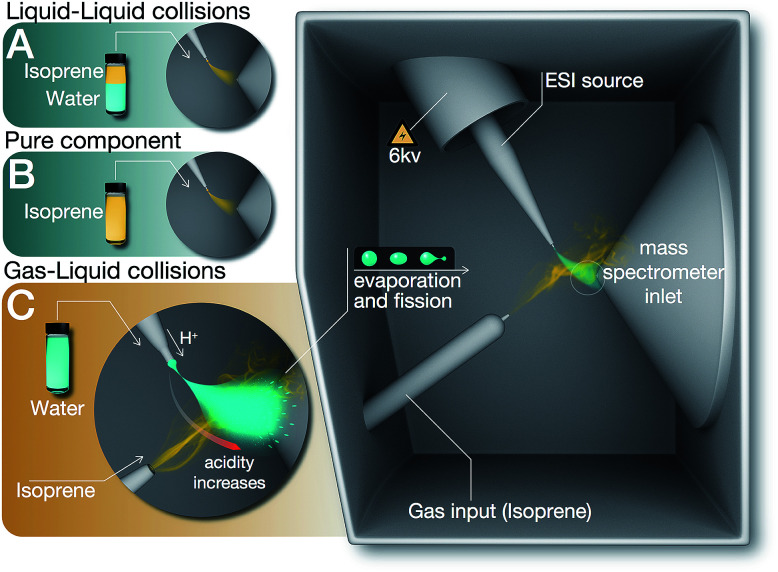
Summary of the experiments (A), (B), and (C) reported in this work. (A) Liquid–liquid collisions: mixtures of isoprene, pH-adjusted water, and air in the volumetric ratio 1 : 6 : 3 was agitated at 1200 rpm (for 6, 60, 360 minutes) followed by ESIMS analysis of the organic phase. (B) As-purchased liquid isoprene was injected directly in the ESIMS. (C) Gas–liquid collisions: electrosprays of water (pH range 1–13) were collided with a stream of air carrying isoprene gas, followed by mass spectrometric detection (Methods).

As delineated in Fig. 1 and summarized in Table 1, we report on the following sets of ESIMS (detection limit = ∼1 nM) and ^1^H-NMR (detection limit = ∼10 μM) experiments:

**Table 1:** Experimental summary

	(A) Liquid–liquid collisions	(B) Pure components	(C) Gas–liquid collisions
	Water(L)–isoprene(L)	Isoprene(L), acetone(L), ethanol(L)	Isoprene(G)–water(L)
**ESIMS**	Organics injected	Components injected	Water injected
pH	1–13	—	1–13
pNaCl	—	—	1–9
Shaking time	6, 60, 360 min	—	—
Voltage	6 kV	6 kV	6–8 kV
Capillary temperature	150 °C	30–330 °C	150 °C
^**1**^ **H-NMR**	Organics from (**A**) and condensed vapors (**A1**)	Isoprene(L)	—
pH	1.5	—	—
Shaking time	6, 60, 360 min	—	—
Aqueous phase	D_2_O, H_2_O	—	—

### (A) Liquid–liquid collisions

At NTP, we combined liquid isoprene with pH-adjusted H_2_O or D_2_O, 1 ≤ pH ≤ 13, in a volumetric ratio 1 : 6 : 3 (isoprene : water : air), agitated the emulsions at 1200 rpm in a vortexer for 6, 60, or 360 minutes, and analyzed the organic phases after phase-separation by ESIMS and ^1^H-NMR. Since the air in the reaction vessels was saturated with isoprene, those experiments also ensured the presence of the products of reactions between the gas-phase isoprene and pH-adjusted water in the organic phase.

#### (A1) Condensed vapors from electrosprays of organic phase from (A)

After the liquid–liquid collision reactions (A) were over and the organic phases were electrosprayed in ESIMS for characterization, we condensed the sprays and analyzed them by ^1^H-NMR. The ^1^H-NMR-based investigation of the reaction products from experiments (A) before and after electrospraying was carried out to pinpoint the effects, if any, of electrospraying on the formation of the products.

### (B) Pure components

We analyzed as-purchased isoprene, acetone, and ethanol by ESIMS and ^1^H-NMR.

### (C) Gas–liquid collisions

We created electrosprays of aqueous solutions with varying ionic strengths and pH, and intersected them with a stream of gas-phase isoprene (0.48 g min^−1^ carried by N_2_ gas flowing at 600 mL min^−1^, *i.e.* isoprene gas concentration was 800 mg L^−1^) followed by mass spectrometric detection (Methods).

Hereafter, throughout the paper, we will refer to our experiments on the liquid–liquid collisions as (A), condensed vapors from the electrosprays (A1), pure isoprene as (B), and gas–liquid collisions in the ESIMS as (C) (Fig. 1, Table 1).

**Fig. 2:**
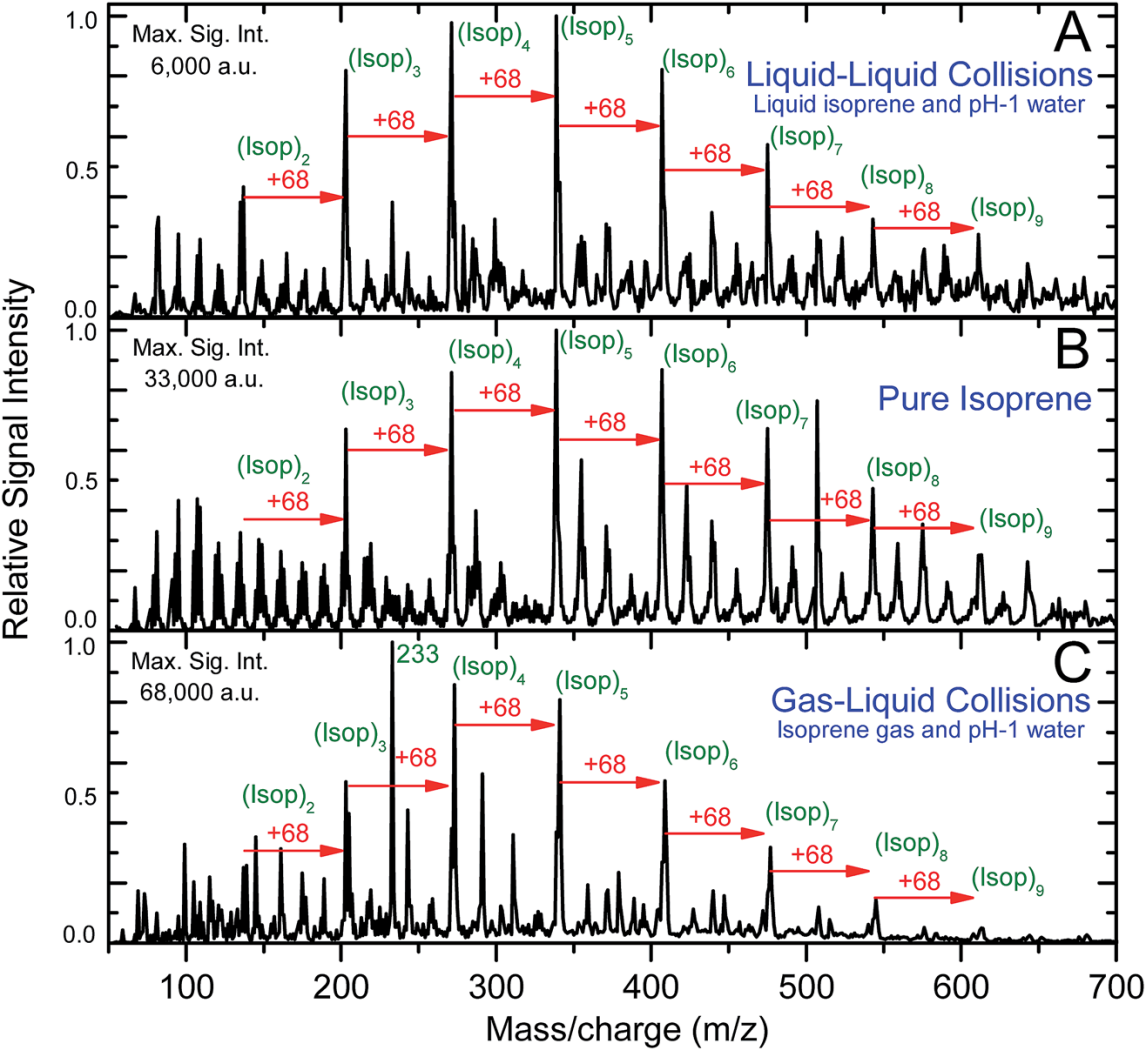
ESIMS spectra for sets of experiments A, B, and C: the dominant peaks correspond to protonated oligomers of isoprene, [(Isop)_*n*_·H]^+^, and the secondary peaks correspond to fragments of the isoprene molecules bonded to the primary oligomers. (A) ESIMS spectra of the organic phase from the emulsion of liquid isoprene in water at pH = 1 and air (1 : 6 : 3 v/v/v) that was agitated at 1200 rpm for 360 minutes. (B) ESIMS spectra of as-purchased liquid isoprene. (C) ESIMS spectra of products of gas–liquid collisions between water (pH = 1) and gas-phase isoprene (Methods).

Intriguingly, the ESIMS spectra from the above-mentioned experiments (A) at pH = 1, (B), and (C) at pH = 1 were nearly identical after normalizing with the maximum intensity (Fig. 2A–C and Section S_a_†). The positions of the main peaks in the mass spectra fitted the general formula, [(Isop)_*n*_·H]^+^, which corresponded to covalently bonded oligomers of isoprene with one excess proton. In Section S_b_ and Fig. S1† we present the evidence proving that the peaks did not correspond to physisorbed clusters. In experiments (A), the mass spectra remained the same as the duration of mixing varied from 6 min to 6 h (Fig. S2†). We also observed numerous secondary peaks between the primary [(Isop)_*n*_·H]^+^ peaks. While a detailed characterization of those peaks falls beyond the scope of this work, we speculate that they are composed of C1–C4 fragments covalently bonded with the [(Isop)_*n*_·H]^+^ oligomers, for example *m*/*z* = 233 (Fig. 2C and S3†).

**Fig. 3:**
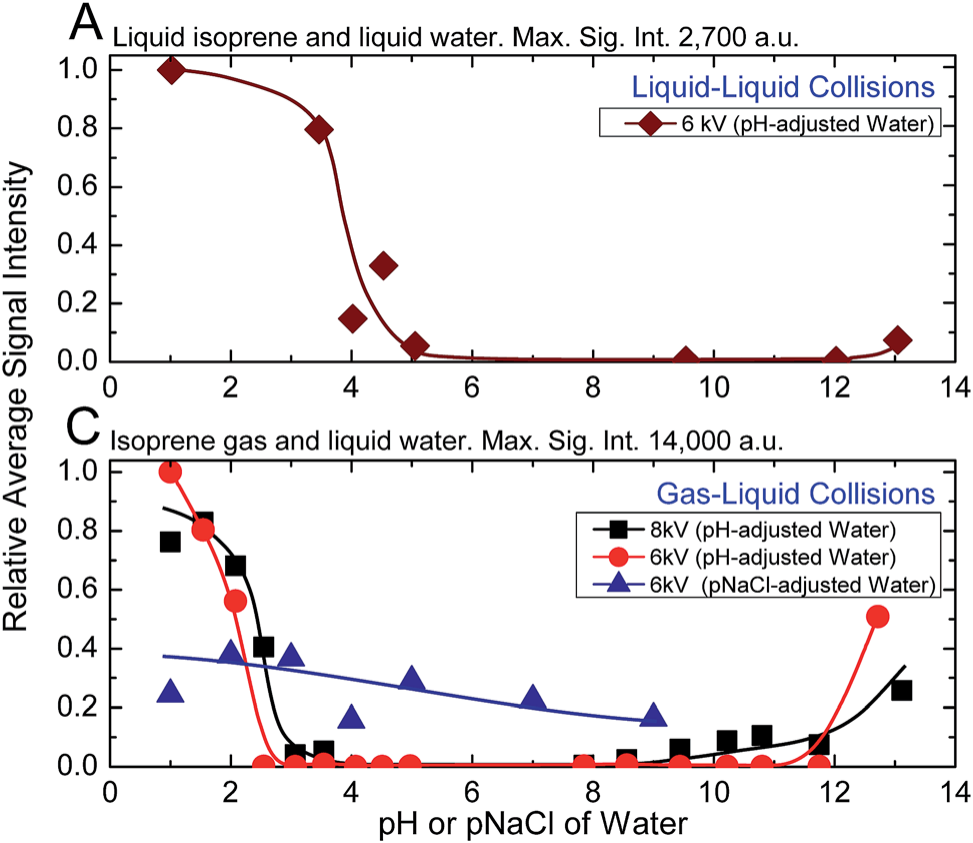
Influence of the ionic strength and ESI voltage on the ESIMS spectra of experimental sets (A) and (C). On the *y*-axis, we plot the average mass spectral intensity of all the oligomeric peaks [(Isop)_*n*_·H]^+^, given by 
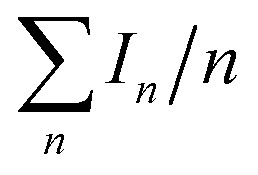
, normalized by the highest datum in each plot. (A) Liquid–liquid collisions: the ESIMS spectra demonstrated protonation and oligomerization of isoprene after emulsions of isoprene in water with pH ≤ 3.6 and pH > 12 and air in a 1 : 6 : 3 ratio (v/v/v) were agitated at 1200 rpm for 360 minutes. (C) Gas–liquid collisions: the ESIMS spectra demonstrated protonation and oligomerization of isoprene gas after collision with electrosprays of water with pH ≤ 3.6 and pH > 12, and pH-neutral salty solutions. Curves are added to the plots to aid visualization.

Next, we investigated the role of water pH on the oligomerization of isoprene in experiments (A) and (C). When the products were characterized by ESIMS, we noticed that the oligomers [(Isop)_*n*_·H]^+^ appeared when the aqueous phase had pH ≤ 3.6 (Fig. 3). Those observations have been reported previously^18,40,41,46,47^ and ascribed to the superacidity of the air–water interface at pH ≤ 3.6. However, we also found that the ESIMS spectra from both experiments, (A) and (C), yielded oligomers [(Isop)_*n*_·H]^+^ for the acidic, basic, and pH-neutral salty solutions (Fig. 3; compare Fig. 2A and C with Fig. S3† panels C1, C2, and C3). We note that for electrosprays produced from pH > 7 water and salty water, counterions, such as Na^+^, could influence the fate of reactions, but we have not investigated those factors.

**Fig. 4:**
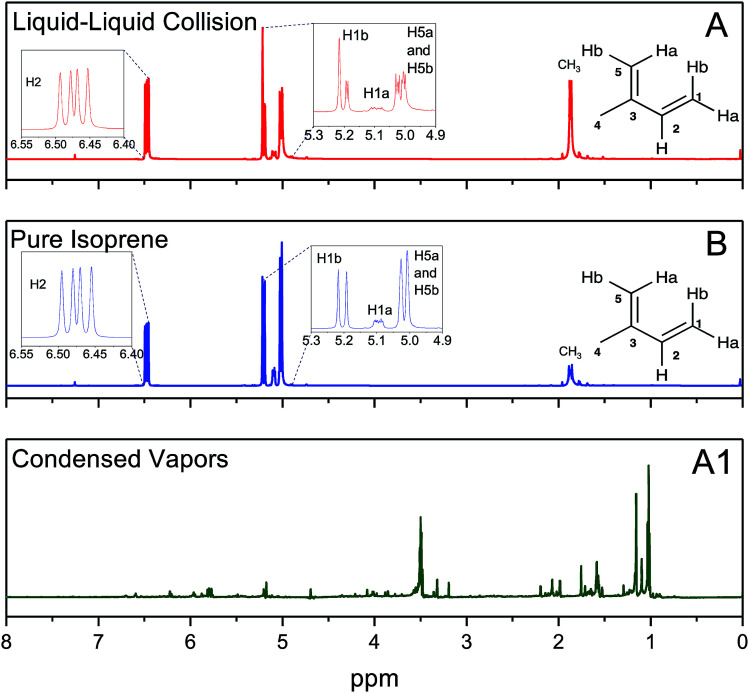
(A) ^1^H-NMR spectra of the organic phase after shaking liquid isoprene with pH 1.5 water and air in a volumetric ratio of 1 : 6 : 3 for 60 minutes. (B) ^1^H-NMR spectra of as-purchased isoprene. (A1) ^1^H-NMR spectra of the condensed exhaust from the electrosprays of the organic phase after the liquid–liquid collision (A) experiments. The nearly identical spectra for experimental sets (A) and (B) demonstrate that there was no detectable oligomerization of isoprene during the vigorous shaking of emulsions comprised of liquid isoprene with pH 1.5 water and air in a volumetric ratio of 1 : 6 : 3 (NMR resolution ∼10 μM).

In experiments (A), after the emulsions comprising liquid isoprene, liquid water at pH = 1.5, and air (containing saturated gaseous isoprene) were vigorously mixed (for 6 min, 60 min, and 360 min) we compared the organic layers after phase separation by ^1^H-NMR. We also recorded the ^1^H-NMR spectra of pure, as-purchased isoprene (B). To our surprise, the ^1^H-NMR spectra from all of the set (A) samples were identical to those of set (B), indicating that the effect of the duration of shaking (6–360 min), the pH (1–13), the isotope (H_2_O *versus* D_2_O) and the presence of gaseous isoprene colliding with pH-adjusted water did not lead to any oligomers within the detection limit of 10 μM (Fig. 4A and B). To investigate further, we condensed the vapors from the ESIMS exhaust (A1) after injecting the set (A) samples (1 ≤ pH ≤ 13), and obtained their ^1^H-NMR spectra. All of (A1) samples showed spectra similar to each other (Fig. 4A1). The ^1^H-NMR spectra of (A) and (B) showed no sign of oligomers in the products: they contained a singlet at 1.87 ppm due to the resonance of the 3 protons in CH_3_; three dublets at 5.02 ppm due to the resonance of the two protons H5b and H5b (coupling constant, *J* = 13.2 Hz), 5.09 ppm due to the resonance of H1a with a *cis*-coupling constant, *J* = 10.8 Hz; a dublet at 5.20 ppm due to the resonance of H1b with a *trans*-coupling constant of *J* = 17.5 Hz; and two dublets at 6.47 ppm due to the resonance of the protons H2a and H2b with the corresponding *trans*- and *cis*-coupling constants, *J* = 17.5 Hz and *J* = 10.8 Hz. In contrast, the ^1^H-NMR spectra of the condensed vapors from the electrosprays (A1) of the organic phase after the liquid–liquid collisions (A) demonstrated a dramatic increase in the complexity of the spectrum,^74^ indicating that the protonation and oligomerization of isoprene took place exclusively in the electrosprays.

## Discussion

Our investigation of experiments (A) with ^1^H-NMR revealed that a significant enhancement in the hydrophobe–water surface area was not sufficient for observable rate accelerations in emulsions of isoprene (gas and liquid) with pH-adjusted water at NTP conditions. On the other hand, analysis of experiments (A), (B), and (C) by ESIMS and experiments (A1) with ^1^H-NMR unambiguously demonstrated that the chemical reactions took place exclusively in aqueous electrosprays – the acidity, basicity, and saltiness of water all promoted the reactions. Further, as the capillary voltage was increased from 6 kV to 8 kV, the inflection points in experiments (A) and (C) shifted such that the oligomers [(Isop)_*n*_·H]^+^ were detected at lower ionic strengths (Fig. 3C). Collectively, these findings contradict previous claims of ‘superacidity’ of pristine air–water interfaces at pH ≤ 3.6.

Next, we sought to identify the mechanisms underlying the protonation and oligomerization of isoprene in electrosprays of mildly acidic water (experiments C). As discussed above, a variety of parameters could influence reactions therein, including electrical voltage, salts, pH, electrochemical reactions, concentration of reactants in rapidly evaporating drops, and gas-phase reactions.^34–36,44,50,63,64,75–79^ Interestingly, by monitoring the changes in the surface tension of pendant water drops exposed to isoprene gas, we found that gas-phase isoprene molecules could adsorb at the air–water interface under NTP conditions (Fig. S4†). While the adsorption of non-polar molecules at the air–water interface might appear unexpected, similar phenomenon at the macroscale, entailing the adsorption of hydrophobic particles onto water drops of size 10^−3^ m in air forming ‘liquid marbles’ is well known.^80^ Thus, gas-phase isoprene molecules (partial pressure in our chamber: 0.28 atm) may adsorb onto the positively charged aqueous electrosprays comprising excess protons.^62^ From this stance, three potential mechanisms for the oligomerization of isoprene emerge, which we discuss and evaluate based on our experimental results and quantum mechanical predictions: mechanism M_1_ – the adsorption of isoprene molecules onto the electrosprays increases their concentration at the interface, leading to reactions under the influence of high electric fields, similarly to the oligomerization of pure liquid isoprene on injection into ESIMS (Fig. 2B); mechanism M_2_ – continuous evaporation of positively charged electrosprays renders them increasingly acidic, akin to 50% H_2_SO_4_ solutions,^48^ which drives the liquid-phase oligomerization of the adsorbed isoprene molecules (Section S_c_ and Fig. S5†); mechanism M_3_ – high capillary voltages electrolyze mildly acidic water and the positive charge ensures that the electrosprayed drops contain excess protons that, during Coulomb explosions, eject highly reactive water clusters that protonate and oligomerize isoprene molecules in the gas phase. In the gas phase, strictly, protonation takes place if the proton affinity of the donor, *e.g.*, H_2_O (165 kcal mol^−1^ at NTP), is lower than that of the acceptor, *e.g.*, Isop (197 kcal mol^−1^ at NTP).^81^ This observation has been extensively discussed by Enke and co-workers in the context of water-alcohol mixtures,^82^ among others;^64,77^ these proton–transfer reactions also underlie the basis of the Proton Transfer Reaction Mass Spectrometry (PTRMS) that has been used to detect trace gases in the atmosphere.^77,83^

In all those mechanisms, the initial ionic strength of water (acidic, basic, or salty) and electrical voltage were crucial for the formation of a stable stream of charged microdroplets – the higher the ionic strength of solutions, the lower the requirement for the electrical voltage.^35,36,84–86^ In fact, due to the electrochemical reactions at the electrospray needle under the influence of high electric fields, the electrosprayed droplets from a positively charged capillary should contain more positive ions than in the bulk^58,63,85,87,88^ (Section S_c_, Fig. S5†). Interestingly, for pH-adjusted water electrosprayed at 6 kV, we detected oligomers (Fig. 3C) when pH ≤ 3.6 or pH > 12, whereas for the NaCl solutions, we observed oligomers at concentrations as low as 10^−9^ M (pNa = 9). Yet, the higher intensities of the [(Isop)_*n*_·H]^+^ at pH ≤ 3.6 in comparison to the salty solutions (Fig. 3C) indicate that the proposed mechanism M_1_ is unlikely to play a crucial role in the case of gaseous isoprene interacting with electrosprays of water.

Following our logical exclusion of mechanism M_1_, we are left with mechanisms M_2_ and M_3_, *i.e.* did the reactions take place on the surface of electrosprayed water droplets or in the gas-phase? Whether or not the electrospray spectra represent the solvent- or gas-phase chemistries/characteristics is a much-debated matter and case-specific.^36,38,76,89,90^ Obviously, the answer would have a bearing on the questions (ii–iv) outlined above, because the kinetics and thermodynamics of reactions in bulk and gas-phase differ dramatically.^21^ Recently, Silveira and co-workers employed cryogenic ion mobility mass spectrometry to demonstrate the effects of rapid dehydration on the structures of undecapeptite substance during the final stages of electrospray ionization.^90^ To further clarify the role of hydration on the protonation and oligomerization of isoprene in our experiments with mildly acidic water (electrosprays and emulsions), we carried out quantum mechanics calculations.

## Computational calculations

To gain qualitative molecular-scale insights into our experiments with mildly acidic water, we carried out density functional theory (DFT) calculations (Computational methods). M06, a hybrid meta-generalized gradient approximation (*meta*-GGA) functional, is known to provide an accurate description of the ground-state thermochemistry, thermochemical kinetics, and transition state structures and energies for a wide series of organic reactions.^91–96^ We have previously confirmed that the binding energies of water clusters, (H_2_O)_*n*_ (range *n* = 2–8, 20), along with the hydration and neutralization energies of hydroxide and hydronium ions using DFT functionals (M06, M06-2X, M06-L, B3LYP, X3LYP) are in excellent agreement with the high-level theory (CCSD(T)/aug-cc-p VDZ level), both with and without the basis set superposition error correction.^91^ For instance, our *ab initio* predictions of the proton transfer thermodynamics between H_3_O^+^(g) and Isop(g) was Δ*G*^0^ = −30.7 kcal mol^−1^, in accordance with the difference in the experimental gas-phase basicity (GB) of H_2_O (GB_H2O_ = −157.7 kcal mol^−1^) and Isop (GB_ISO_ = −190.6 kcal mol^−1^), ΔGB = −32.9 kcal mol^−1^ (Fig. S6†).^97^ Furthermore, the *trans*- or *cis*-Isop(g) spontaneously added onto (Isop·H^+^ + H_2_O), leading to cyclic (Δ*G*^0^ = −40 kcal mol^−1^) or acyclic monoterpenes (Δ*G*^0^ = −9 kcal mol^−1^), (Fig. S7 and S8†), as also noted by other researchers.^48,98^ Next, we calculated the kinetic barriers for protonation and oligomerization of Isop on a small water cluster containing an extra proton, (H_2_O)_3_·H^+^, as representative of our electrospray experiments and a larger cluster, (H_2_O)_36_·H^+^, representing the pristine air–water interface. The sizes of the clusters were guided, in principle, by previous experimental and theoretical work on the hydration of protons,^99–101^ showing the asymptotic stabilization of a proton with increasing cluster size, and limited by computational expense. The initial geometry of the smaller cluster was obtained from the Cambridge Cluster Database,^102^ while the larger cluster geometry was obtained from an SPC/E bulk water box equilibrated at 298 K and at 1 atm pressure yielding the bulk density of 1 g cm^−3^. We took a cluster of 36 water molecules from the equilibrated water box as a surrogate for the air–water interface. We added a proton to this cluster and applied Born–Oppenheimer Molecular Dynamics (BOMD) simulations (Computational methods) to obtain various low-energy conformers (Table S2, Section S_d_†). We chose a cluster with the proton on the surface that facilitated the subsequent study of the activation barriers through DFT calculations (Computational methods).

**Fig. 5:**
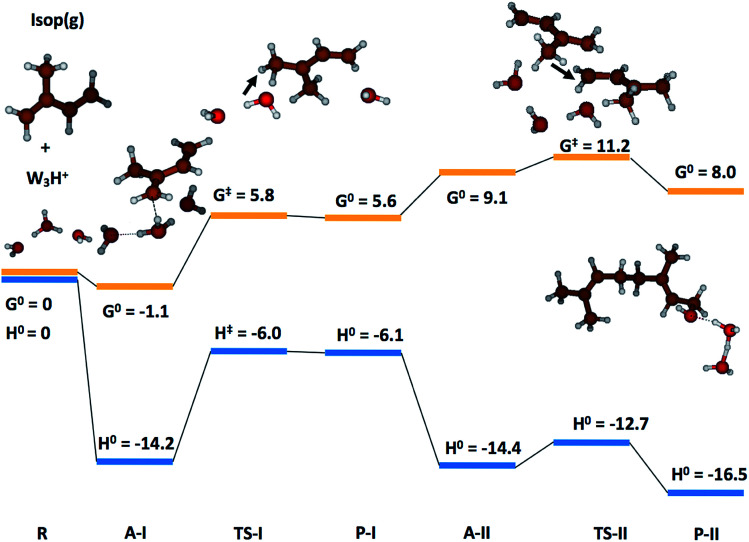
Quantum mechanics-based free energy and enthalpy landscapes for protonation and oligomerization of isoprene while interacting with a cluster comprising three water molecules and one excess proton, (H_2_O)_3_·H^+^. Due to its incomplete hydration (compared to bulk), the proton exhibited extreme acidity. The free energy barrier for the proton transfer from (H_2_O)_3_·H^+^ to isoprene(g) was Δ*G*^‡^ = 6.9 kcal mol^−1^ and the barrier to subsequent oligomerization with another free isoprene(g) was Δ*G*^‡^ = 2.1 kcal mol^−1^, which are easily surmountable under ambient NTP conditions within the timescale of our experiments (∼1 ms). These model predictions support mechanism M_3_.

**Fig. 6:**
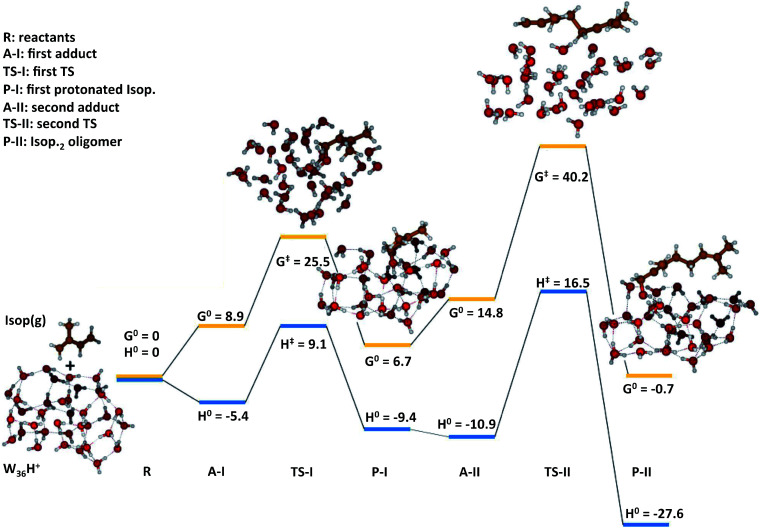
Quantum mechanics-based free energy and enthalpy landscapes for protonation and oligomerization of isoprene on a cluster consisting of 36 water molecules and an excess proton, (H_2_O)_36_·H^+^, representative of very small water droplets. The kinetic barriers preventing proton transfer to isoprene and its subsequent oligomerization were Δ*G*^‡^ = 25.5 kcal mol^−1^ and Δ*G*^‡^ = 40.2 kcal mol^−1^ respectively, which were insurmountable under ambient NTP conditions within the timescale of our experiments (∼1 ms). The predictions of this model suggest that the reactions of isoprene in electrosprays cannot involve liquid-phase drops. These predictions also support the proposed mechanisms M_3_ by ruling out the possible reactions through M_2_.

In the case of (H_2_O)_3_·H^+^, representative of electrosprays, the proton was extremely reactive/acidic due to insufficient hydration – the incipient isoprene molecule (Isop) fell into a shallow potential well forming an adduct (Fig. 5). The free energy barrier for proton transfer from (H_2_O)_3_·H^+^ to Isop(g) was Δ*G*^‡^ = 6.9 kcal mol^−1^, and the barrier to the oligomerization with another free Isop(g) was Δ*G*^‡^ = 2.1 kcal mol^−1^ (Fig. 5). On the other hand, the predictions for those barriers for protonation and oligomerization of isoprene with the larger water cluster, (H_2_O)_36_·H^+^, representative of the air–water interface, were Δ*G*^‡^ = 25.5 kcal mol^−1^ and Δ*G*^‡^ = 40.2 kcal mol^−1^, respectively, which are insurmountable under ambient conditions within a 1 ms time-frame (Fig. 6). The stark differences in the free energy landscapes for the interactions of Isop(g) with minimally hydrated hydronium ions, available in electrosprays, and larger water clusters can simply be understood in terms of the enthalpies of hydration of gas-phase protons that decrease monotonically with the addition of water molecules;^99–101^ the proton activity also decreases commensurately and, in fact, is expected to be even lower for the air–water interface than in the (H_2_O)_36_·H^+^ cluster.

To test the effects of larger basis-sets on these predictions, we compared the results of our M06/6-311+G*/6-311++G** calculations with M06/6-311++G** for the smaller cluster, and found very similar results (Section S_d_, Table S1†). Towards further benchmarking, we carried out single point energy calculations on the structures optimized at the M06/6-311++G**-level with the CCSD(T)-level theory,^66^ and found that the qualitative trends in the reaction kinetics remained unchanged (Table S1†). We note that we could only perform the benchmarking for the smaller system due to the computational expense. Thus, we expect that the relative differences in the barrier heights for the reactions are unlikely to change significantly between DFT/M06 and CC for our systems. Besides, our ^1^H-NMR experiments present unambiguous evidence for these predictions. To summarize, our calculations demonstrate that the kinetic barriers for the protonation and oligomerization of isoprene are easily surmountable in the smaller acidic cluster (available in electrosprays only) and prohibited in larger acidic clusters at 298 K and 1 atm (a surrogate for the pristine air–water interface).

## Conclusion and outlook

Based on our experimental investigation of oil–water and air–water interfaces of isoprene with pH-adjusted water, analyzed by ESIMS and ^1^H-NMR along with quantum mechanical predictions, we address the questions outlined in the introduction as follows:

(i) Aqueous electrosprays do not always report on the thermodynamic properties of pristine air–water interfaces.

(ii) The observed chemical reactions of isoprene in aqueous electrosprays were not driven by the enhancement in the hydrophobe–water interfacial area, as evidenced by the lack thereof in vigorously mixed emulsions of isoprene and pH-adjusted water. Thus, the mechanisms underlying the ‘on-water’ catalysis^54–57^ must be different from those leading to rate accelerations in the aqueous electrosprays.^103^ Electrosprays of water must facilitate additional chemical pathways, such as reactions with partially hydrated (gas-phase) hydroniums, which are not accessible in vigorously mixed oil–water emulsions or pristine aqueous interfaces.

(iii) Reactions of isoprene in aqueous electrosprays were driven by non-equilibrium conditions therein – most importantly, due to the rapid evaporation of water leading to highly concentrated droplets and partially hydrated hydronium ions (mechanism M_3_). It is, thus, crucial to distinguish their contribution from purely ‘interfacial effects’ towards dramatic rate enhancements in aqueous electrosprays.^50,53,64,78,104,105^

(iv) Gas-phase reactions could play a significant role in the electrosprays – in our experiments, reactions between partially hydrated protons and isoprene led to its protonation and oligomerization, as recently suggested by Yan & co-workers^53^ and demonstrated by Jacobs & co-workers.^106^

Our experimental and theoretical results demonstrate that chemistries in aqueous electrosprays do not necessarily correspond to those at the pristine air–water interface and oil–water emulsions at NTP. These findings also contradict the previous claims of the superacidity of the pristine air–water interface as the bulk acidity approaches pH ≤ 3.6;^40^ the proposal for the mildly acidic environmental surfaces to act as the primary sink for the atmospheric isoprene/terpenes should also be reevaluated.^41,46,47^ While the potential of aqueous electrosprays to produce high-value products appears promising, those reactions are unlikely to be realized at pristine aqueous interfaces because of the seminal role of the non-equilibrium effects, such as the formation of water clusters with minimally hydrated hydronium ions.^49,78,106^ We do note that air–water interfaces could, perhaps, be investigated semi-quantitatively through electrosprays, if the reactants do not participate in gas-phase, acid catalyzed, or redox reactions therein^51,107–109^ and/or the gas-phase reactants do not dissolve in the droplets to re-emerge as interfacial species; a careful case-by-case assessment is needed. The mechanisms underlying the protonation and oligomerization of isoprene gas on electrosprays of pH > 7 water also warrant further investigation. We conclude by stressing on the importance of combining complementary experimental techniques, *ab initio* calculations and molecular dynamics simulations in the quest to unravel phenomena occurring at the pristine air–water interface.

## Conflicts of interest

There are no conflicts to declare.

## Supplementary Material

SC-010-C8SC05538F-s001
