# Supplementary material for: The chemical reactions in electrosprays of water do not always correspond to those at the pristine air–water interface
Source: Chem Sci. 2018 Dec 21;10(9):2566–77. doi: 10.1039/c8sc05538f (PMC6422012; doi:10.1039/c8sc05538f)
Supplement: SC-010-C8SC05538F-s001 [file SC-010-C8SC05538F-s001.pdf]

Electronic Supplementary Material (ESI) for Chemical Science.
This journal is © The Royal Society of Chemistry 2019

The Chemical Reactions in Electrosprays of Water Do *Not* Always Correspond to Those at the Pristine Air-Water Interface

Adair Gallo Jr. [1,2,3], *Andreia S. Farinha*[1,2,3], *Miguel Dinis*[1,4], *Abdul-Hamid Emwas*[1,5], *Adriano Santana*[1,2,3], *Robert J. Nielsen*[6], *William A. Goddard III*[6], *Himanshu Mishra*[1,2,3,*]

[1]King Abdullah University of Science and Technology (KAUST),
[2]Water Desalination and Reuse Center (WDRC),
[3]Division of Biological and Environmental Sciences (BESE),
[4]KAUST Catalysis Center (KCC),
[5]Imaging and Characterization Core Laboratory,
Thuwal 23955-6900, Saudi Arabia;

[6]Materials and Process Simulation Center, California Institute of Technology,
Pasadena, CA 91125, USA

* Himanshu.Mishra@Kaust.edu.sa

Supporting Information

Section S_a:

Comparison of the different ESIMS signal intensities for $[(Isop)_n.H]^+$ species in experiments (A), (B), and (C) (Figure 2). The observed signal intensities were surprisingly the strongest in the case of the gas-liquid collisions between *Isop*(g) and acidic water (pH = 1) (**C**), followed by pure isoprene (**B**), and the organic phase from emulsions of liquid isoprene and acidic water (pH = 1) (**A**). We consider that during the vigorous shaking of the isoprene-water emulsions, a fraction of the aqueous content was dissolved into the organic phase, i.e. isoprene. Subsequently, as the organic phase was electrosprayed after the phase separation, the aqueous components decreased the intensity of the isoprene oligomers due to the competing effect of the ions being attracted into the mass spectrometer. Since our ESIMS has a minimum detection limit of m/z = 50, we were unable to observe those smaller ions. The higher signal intensities (maximum signal intensity 68,000 a.u.) of the gas-liquid collision (**C**) could be attributed to the considerably higher flow rate of isoprene, 0.48 g/min in 600 mL/min of air, into the atmospheric chamber of the ESIMS, compared to 10 µL/min in experiments (**A**) and (**B**).

Section S_b:

Determining if the mass spectrometric peaks (Figures 2, S3) correspond to covalently bonded oligomers or physisorbed clusters of isoprene. The temperature of the glass capillary at the inlet of the mass spectrometer is an important variable in ESIMS. It provides thermal energy to the incoming droplets, facilitating the evaporation of the solvent or neutral molecules – at elevated temperatures, the non-covalently bonded clusters become unstable and the molecules separate, whereas covalently bonded oligomers survive. Thus, based on the changes in the mass spectral intensities as a function of the glass capillary temperature, we could discern if the peaks comprised of covalently bonded or non-covalently bonded species. For instance, while injecting pure acetone, ethanol, and isoprene (Figure S1 – B1, B2, and B3, respectively), we observed peaks in the spectra corresponding to $(M)_n$ ($m/z = n.M + H^+$), i.e., $(Ace)_2$, $(Et)_2$, $(Et)_3$, and $(Isop)_{2,3,4...}$(Figure S1 – top right corner insets). However, as we increased the temperature of the glass capillary, we noticed that the intensities of the heavier peaks of acetone and ethanol dramatically decreased with increasing temperatures (Panels B1 and B2 in Figure S1). In stark contrast, the mass spectral intensities for isoprene did not vary much as the temperature increased (Panel B3 in Figure S1). Thus, our simple experiment helped us conclude that while acetone and ethanol form non-covalent clusters[1] with an excess proton, isoprene forms covalently bonded oligomers. Our conclusion was further corroborated by the clean spectra of acetone and ethanol, evidencing that negligible fragmentation and reactions were present.

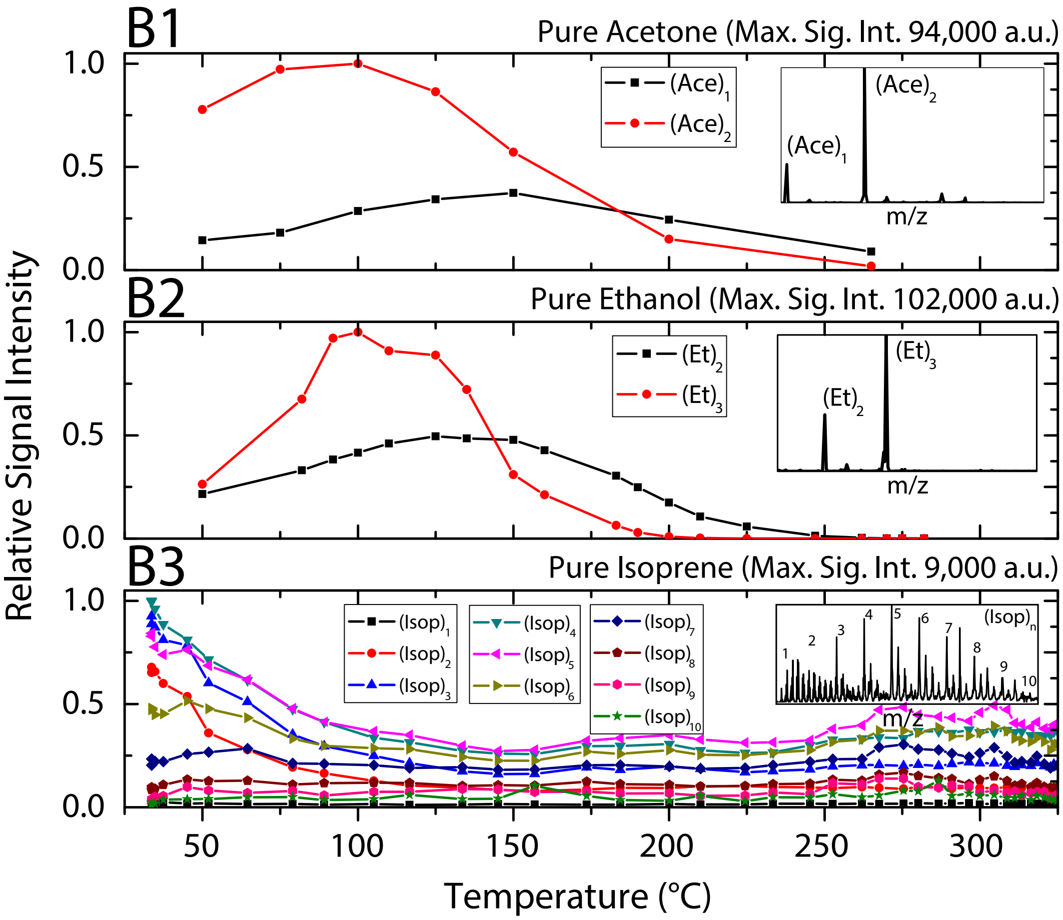

Figure S1 – Influence of capillary temperature on the detection of $[(M)_n{+}H]^+$ peaks at the ESIMS. (**B1**) Comparison between signal intensities of peaks from $(Ace)_1$ (m/z 59 = $MW_{ACETONE}$ + MW_{H^+}) and $(Ace)_2$ (m/z 117 = 2.$MW_{ACETONE}$ + MW_{H^+}). (**B2**) Comparison of peaks from $(Et)_2$ (m/z 93 = 2.$MW_{ETHANOL}$ + MW_{H^+}) and $(Et)_2$ (m/z 138 = 3.$MW_{ETHANOL}$ + MW_{H^+}). (**B3**) Comparison of peaks from $(Isop)_1$ (m/z 69 = 1.$MW_{ISOPRENE}$ + MW_{H^+}) and $(Isop)_{10}$ (m/z 681 = 10.$MW_{ISOPRENE}$ + MW_{H^+}). ESIMS set to positive mode, 6 kV, 10 µL/min for acetone and ethanol, and 5 µL/min for isoprene. The insets in the right corners are characteristic ESIMS spectra for each case.

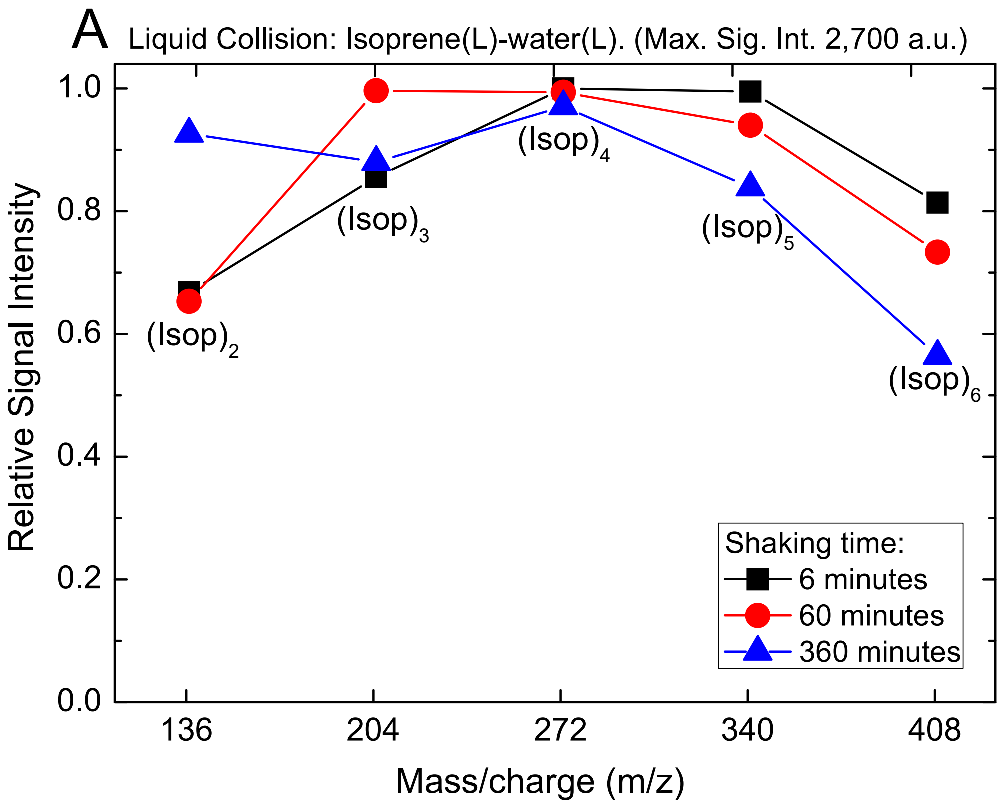

Figure S2 - Influence of shaking duration on the liquid-liquid collisions, experiments (A). Comparison of ESIMS peaks from $(Isop)_2$ (m/z 136 = 1.MW$_{ISOPRENE}$ + MW$_{H^+}$) and $(Isop)_6$ (m/z 409 = 6.MW$_{ISOPRENE}$ + MW$_{H^+}$); ESIMS set to positive mode, 6 kV, 150 °C, 10 μL/min, pH of aqueous phase 1.52. The shaking time did not considerably influence the signal intensity of the liquid-liquid collisions.

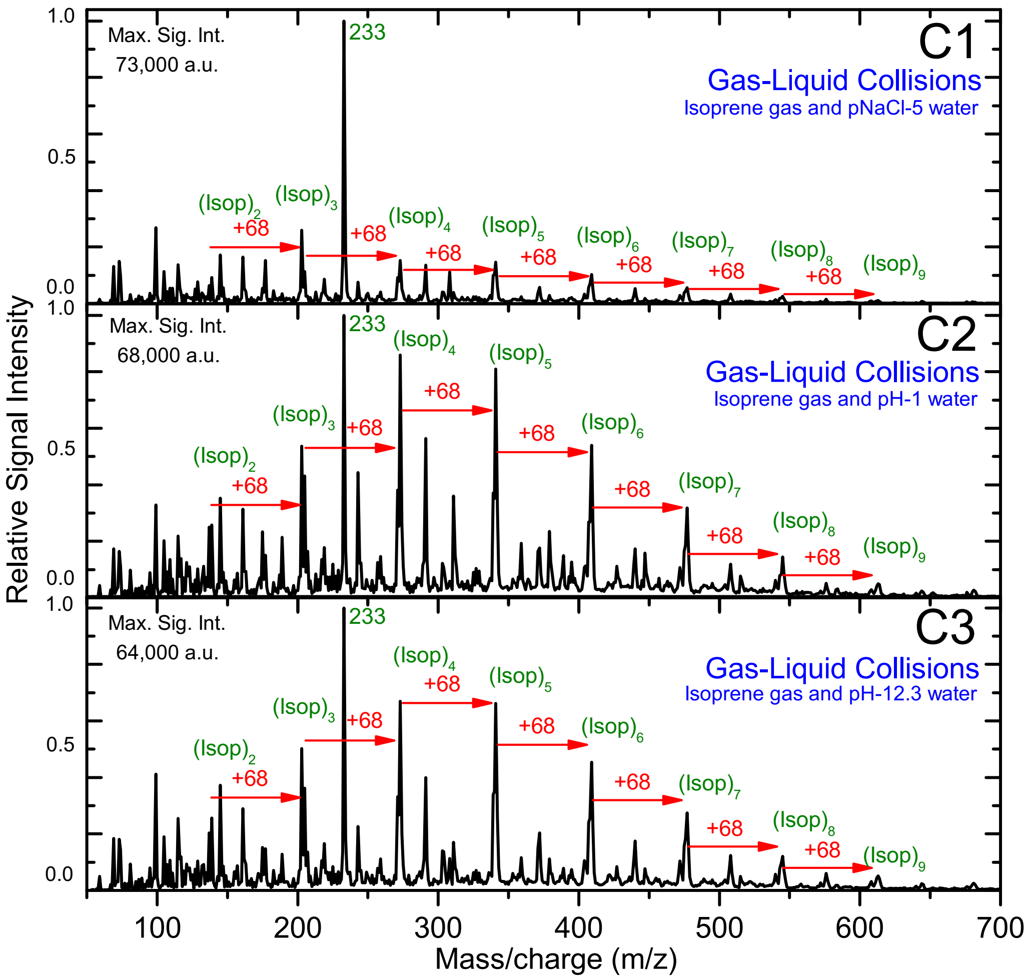

Figure S3 – ESIMS spectra for gas-liquid collisions, experiments (C). (**C1**) Water at p[NaCl] = 5 and flow rate of 1 μL/min; (**C2**) Water at pH = 1 and flow rate of 1 μL/min; (**C3**) Water at pH = 12.3 and flow rate of 1 μL/min. The main peaks correspond to the oligomers of isoprene plus one proton $[(Isop)_n+H]^+$, and are separated by the mass of isoprene (68 *m/z*). The four smaller peaks between the main ones correspond to the four possible fragmentations of the carbon bonds within the isoprene molecule (C_5H_8). In all three cases the stream flow of air (600 mL/min) and gaseous isoprene (which evaporated from the air bubbler at ~0.48 g/min) was directed towards the electrosprayed water jet; the temperature of the capillary in the ESIMS was 150 °C, the electrical potential applied at the electrospray needle was 6 kV, the capillary inlet was grounded, and the separation between the needle and the capillary inlet was ~1 cm.

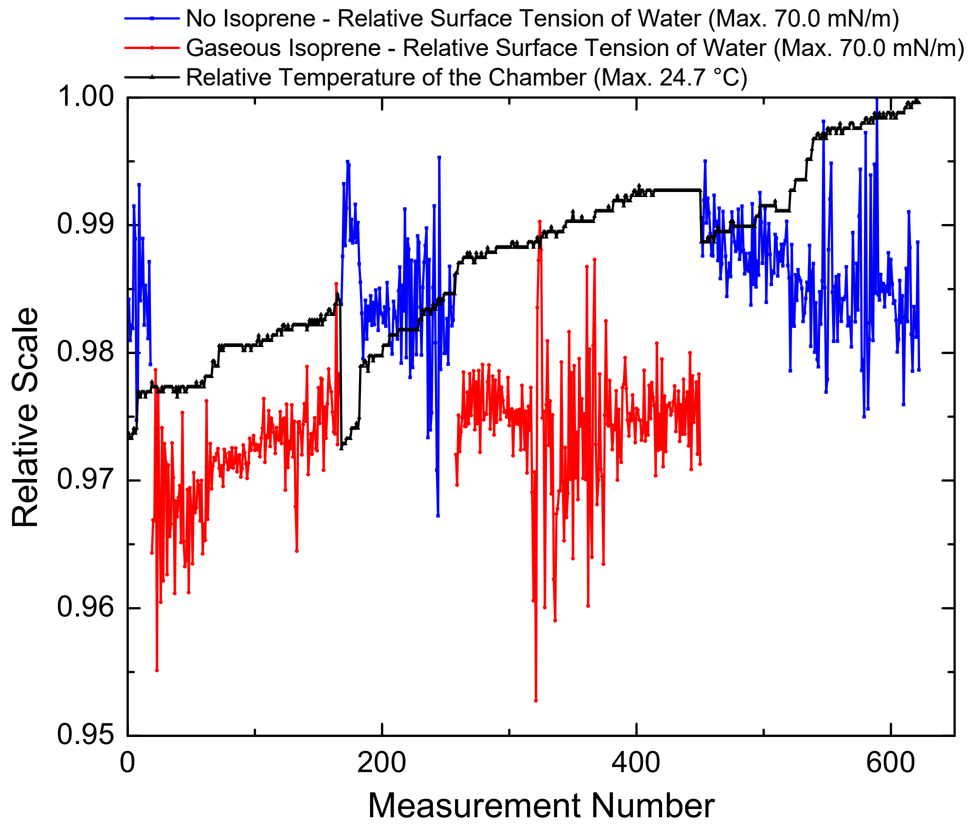

Figure S4 – Influence of gaseous isoprene on the surface tension of a droplet of water. The surface tension was measured by the pendant drop method in a chamber in the presence of air (blue line) and gaseous isoprene in air (red line). The surface tension of the water droplet changed considerably in the presence of gaseous isoprene, indicating adsorption and possibly dissolution of the isoprene into the bulk water.

Section S_c:

What is the role of the net positive charge during droplet formation at the electrospray? Let us consider that the charge separation (and oxidation reactions) at the metallic needle ejecting an analyte under electric fields leads to a decrease in the pH of the just-formed droplets by at least 0.1 (or a 26% increase in the concentration of protons)[2, 3]. The initial droplet size will depend on the electric potential, polarity, needle diameter, ionic strength, surface tension, and viscosity (Figure S5 contains a visual representation of this thought experiment). While evaporating, the charge density of the droplets increases and the Rayleigh limit is eventually reached, i.e. the repulsion of the electrostatics will overpower the cohesion of the surface tension. The excess charge is predicted to be $Q = (k\pi^2\varepsilon_0\gamma R^3)^{1/2}$, where d is the diameter of the droplet at the time of fission, γ is the surface tension of the liquid, ε_0 is the permittivity of a vacuum, and k is a constant[4, 5].

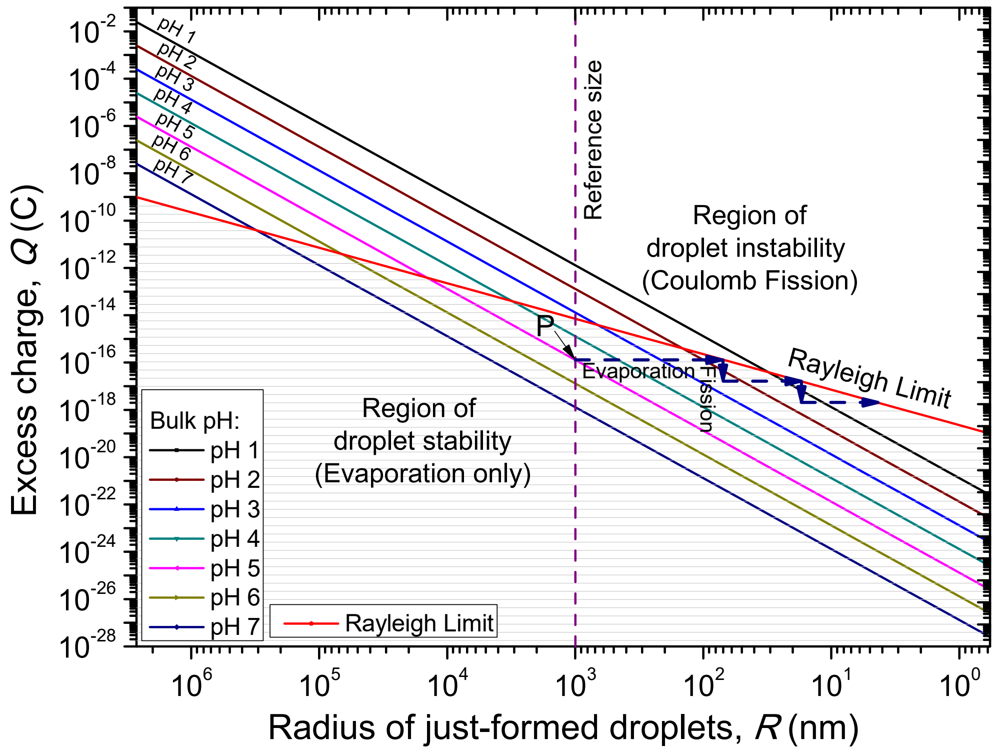

Figure S5 – Simulation of the influence of bulk pH and initial radius on the net/excess charge of electrosprayed droplets. For a given ESI setup, the just-formed droplets will have different diameters depending on experimental conditions such as the electric potential, polarity, needle diameter, ionic strength, and surface tension. We expect that the sooner the droplets reach the Rayleigh limit, the faster they will undergo Coulomb fissions and release highly reactive clusters containing excess hydronium ions. Let us consider water with pH = 5 (pink line): a droplet of 1000 nm is formed with an excess charge of ~26%, i.e. 10^{-16} C (Point *P*). Since this droplet is below the Rayleigh limit, it will not undergo Coulomb fission immediately. However, this pH 5 droplet will evaporate (following the horizontal arrow) and then, after reaching the Rayleigh limit, undergo Coulomb fission. Note that the down arrow representing fission is exaggerated; it shows a ~10-fold decrease in the droplet charge during each fission, while a lower discharge is expected in reality[3]. Conversely, droplets with pH = 1 (black line) would immediately eject hydroniums to the gas phase under same setup conditions. A parallel can be drawn between this process and the proposed Mechanism M_3.

Section S_d:

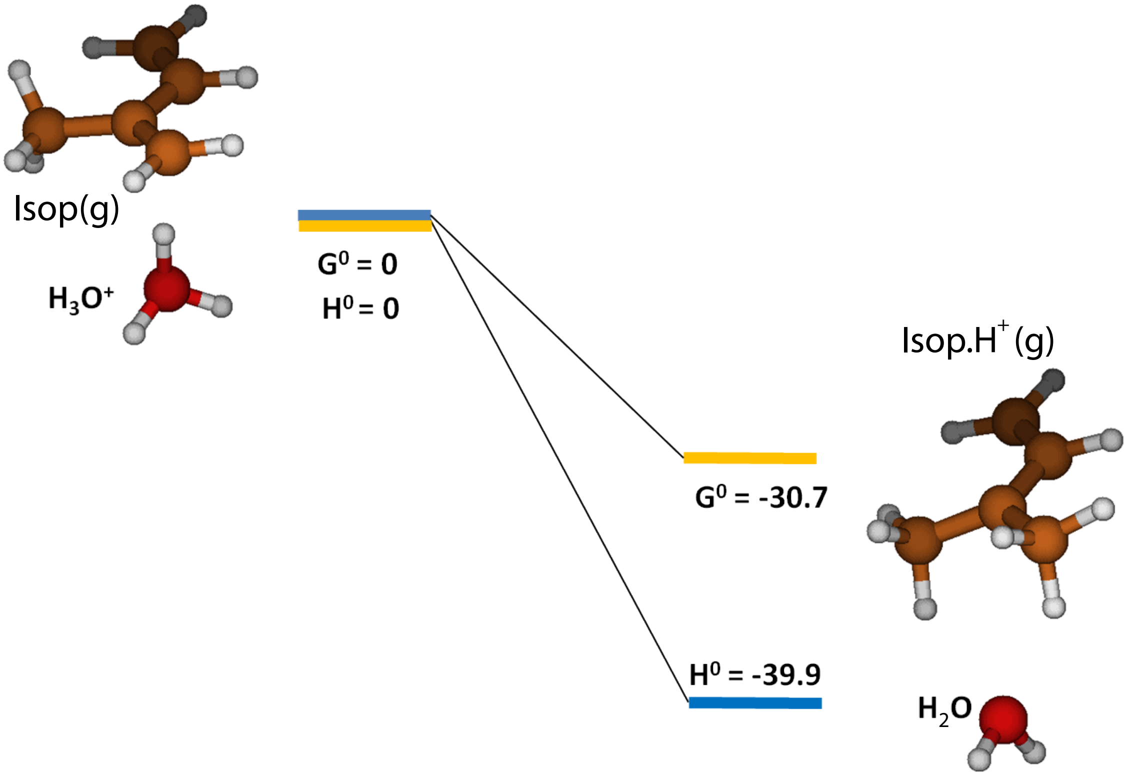

Figure S6 - *Ab initio* predictions of the proton transfer reaction between a gas-phase hydronium ion, H_3O^+(g), and a gas-phase isoprene molecule, *Isop*(g). Theory predicted the reaction to be spontaneous with a free energy change of ΔG^0 = - 30 kcal mol^{-1}, in accordance with the experimental gas-phase basicities (GB) of H_2O (GB_{H2O} = 157.7 kcal mol^{-1}) and *Isop* (GB_{ISO} = 190.6 kcal mol^{-1}); ΔGB = 32.9 kcal mol^{-1}.

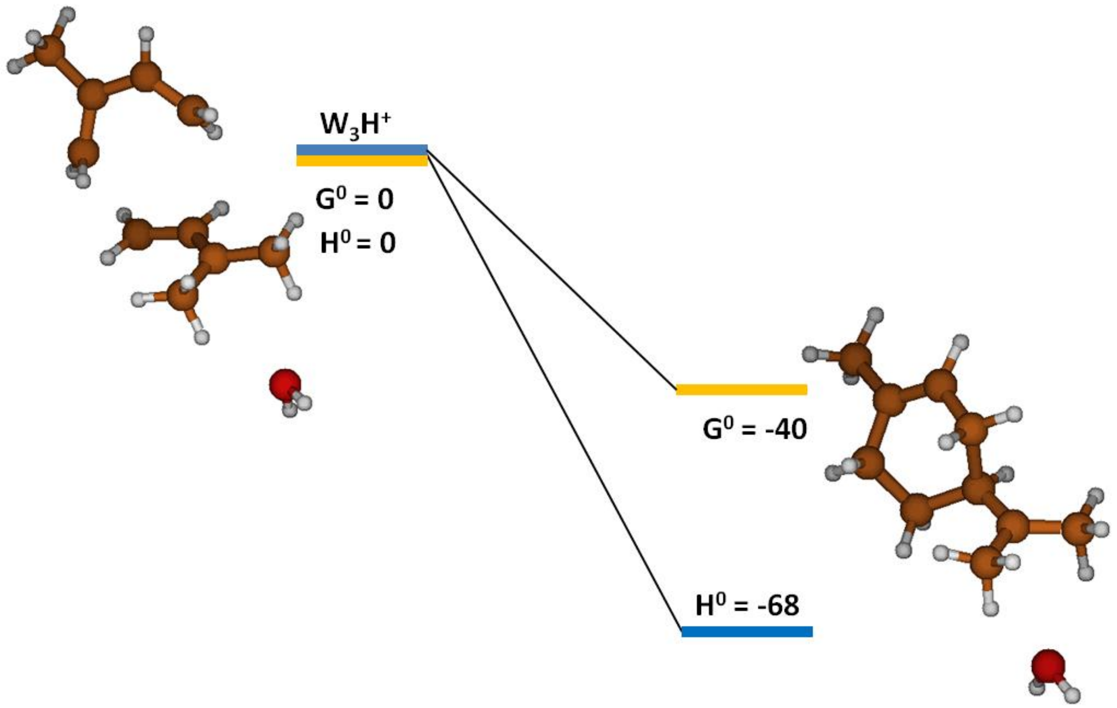

Figure S7 - Spontaneous gas-phase oligomerization of gas-phase cis-isoprene (*Isop*(g)) with a protonated trans-isoprene molecule, leading to a cyclic product. The units for G^0 and H^0 are kcal-mol^{-1}.

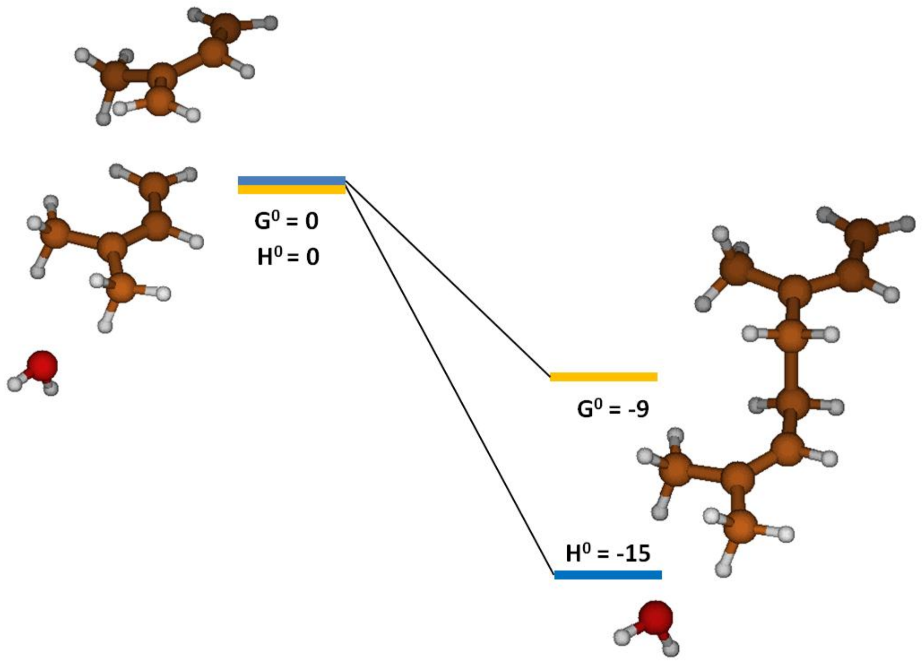

Figure S8 – Spontaneous gas-phase oligomerization of a trans-isoprene (*Isop*(g)) molecule with a protonated trans-isoprene, leading to a linear product. The units for G^0 and H^0 are kcal-mol^{-1}.

Table S1 – Calculated energy landscapes for the interactions/reactions of isoprene with the $(H_2O)_3.H^+$ cluster by three computational approaches with the same functional (M06), but different basis sets:

(A) M06/6-311G* (with single point optimization with 6-311++G,**

(B) M06/6-311++G,**

(C) M06/6-311++G with single-point calculations with CCSD(T)**

STEP	(A) Original calculations (M06/6-311G*/6-311++G**) (kcal/mol)	(B) Revised calculations (M06/6-311++G**) (kcal/mol)	(C) Revised calculations (M06/6-311++G**/CCSD(T)) (kcal/mol)
A-I	-14.2	-14.6	-14.2
TS-I	-6.0	-3.5	-3.7
P-I	-6.1	-4.0	-4.7
A-II	-14.4	-13.7	NA
TS-II	-12.7	-13.3	NA
P-II	-16.5	-20.0	NA

Table S2 – $(H_2O)_{36}.H^+$ snapshots taken from our BOMD simulation at 300K after equilibration for 5 ps (Details in the Computational Methods Section in the manuscript). The cluster size was $\approx 1.5\times 1.3\times 0.8$ nm^3. Due to the small size of the cluster, the proton is always exposed or close to the surface, engaging in hydrogen bonding and breaking. The cluster we employed in our DFT calculations had the proton on the surface to facilitate simpler calculations. Thus, the activation barriers for our calculations with the larger cluster (Figure 6) present the lower bound. This means that the real barriers will be even higher, which further substantiates our point that chemical reactions in electrosprays, proceeding through partially hydrated hydroniums (Figure 5), are kinetically hindered at the pristine air-water interface (Figure 6).

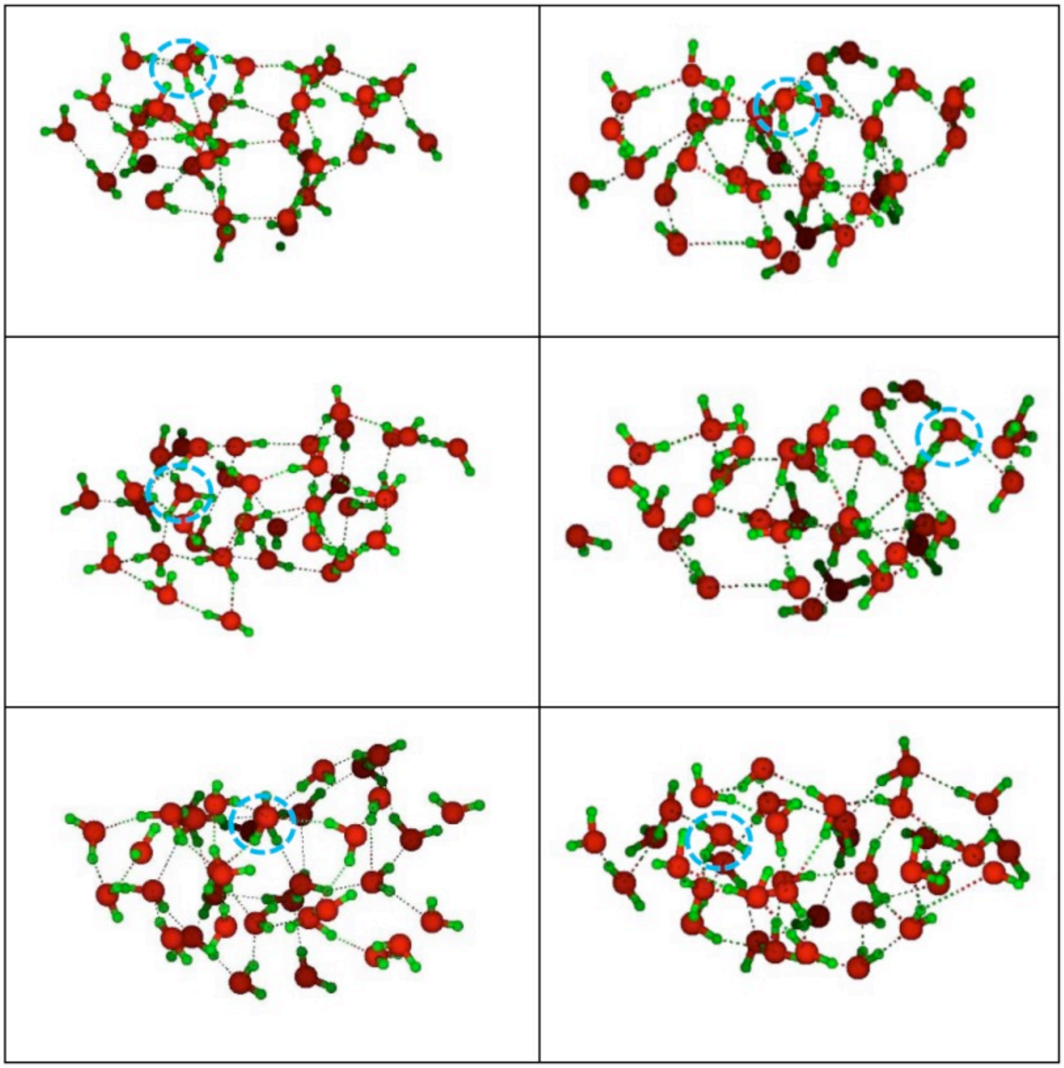

Section S_e:

Description of the NMR: NMR signals showed at the spectra obtained in Liquid-liquid collisions experiments (A) and pure, as-purchased isoprene (B):

^{1}H NMR (700 MHz, Chloroform-*d*) **δ (ppm):** 6.47 (dd, *J* = 17.5, 10.8 Hz, 1H, **H2**), 5.20 (d, *J* = 17.5 Hz, 1H, **H1b**), 5.09 (d, *J* = 10.8 Hz, 1H, **H1a**), 5.02 (d, *J* = 13.2 Hz, 2H, **H5b and H5b**), 1.87 (s, 3H, C**H_3**).

^{13}C NMR (176 MHz, Chloroform-*d*) **δ (ppm):** 142.29 (**C3**), 139.61 (**C2**), 116.76 and 113.65 (**C1** and **C5**), 17.80 (**C4**).

References

1. T. G. Spence, T. D. Burns and L. A. Posey, *The Journal of Physical Chemistry A*, 1997, **101**, 139-144.
2. S. Zhou, B. S. Prebyl and K. D. Cook, *Analytical chemistry*, 2002, **74**, 4885-4888.
3. J. N. Smith, R. C. Flagan and J. L. Beauchamp, *Journal of Physical Chemistry A*, 2002, **106**, 9957-9967.
4. L. Rayleigh, *Philosophical Magazine*, 1882, **14**, 184-186.
5. W. D. Luedtke, U. Landman, Y. H. Chiu, D. J. Levandier, R. A. Dressler, S. Sok and M. S. Gordon, *Journal of Physical Chemistry A*, 2008, **112**, 9628-9649.
6. V. S. Bryantsev, M. S. Diallo and W. A. Goddard, *Journal of Physical Chemistry A*, 2009, **113**, 9559-9567.
7. A. M. Sarotti, A. G. Suarez and R. A. Spanevello, *Tetrahedron Lett.*, 2011, **52**, 3116-3119.
8. Y. Zhao and D. G. Truhlar, *Theor Chem Acc*, 2008, **120**, 215-241.
9. C. P. Kelly, C. J. Cramer and D. G. Truhlar, *J. Phys. Chem. B*, 2004, **108**, 12882-12897.
10. E. P. L. Hunter and S. G. Lias, *J. Phys. Chem. Ref. Data*, 1998, **27**, 413-656.
11. V. B. Kazansky, *Catal. Rev.*, 2001, **43**, 199-232.
12. V. B. Kazansky, *Catalysis Today*, 2002, **73**, 127-137.
